# Supplementary material for: Asynchronous magnetic resonance elastography: Shear wave speed reconstruction using noise correlation of incoherent waves
Source: Magn Reson Med. 2022 Oct 27;89(3):990–1001. doi: 10.1002/mrm.29502 (PMC9792433; doi:10.1002/mrm.29502)
Supplement: Supplementary file 1 — DATA S1 MATLAB code used to generate the numerical simulations of Figure 1 [file MRM-89-990-s002.zip › k-Wave/helpfiles/example_tvsp_snells_law.html]

Snell's Law And Critical Angle Reflection Example (k-Wave)


# Snell's Law And Critical Angle Reflection Example

This example illustrates Snell's law by steering a tone burst from a
linear array transducer within a layered heterogeneous medium. It builds on the Simulating Transducer Field Patterns Example.

- Open the file in the MATLAB Editor
- Run the file in MATLAB

## Contents

- Snell's Law
- Critical Angle Reflection

## Snell's law

It is well known that when a plane wave interacts with a planar interface, the reflected and
transmitted waves will obey Snell's law.

The angle of the incident and reflected waves will be identical, and the angle of the transmitted wave
will vary according to

.

This is simulated here by steering a tone burst from a linear transducer onto
a layered medium heterogeneity. For c1 = 1500 m/s, c2 = 3000 m/s, and an incidence angle of
20 degrees, the transmission angle will be 43 degrees.


## Critical Angle Reflection

When the incidence angle exceeds

the incident wave will be completely reflected. For the parameters used here, this corresponds to
30 degrees. The interaction of the plane wave with the interface for a incidence angle of
35 degrees is shown below. An evanescent wave component that decays quickly further from the
interface is visible, but the planar component of the incident wave has been completely reflected.
